# A machine learning model for predicting lymph node positivity in ovarian cancer: development, validation, and clinical application

**DOI:** 10.3389/fonc.2025.1527674

**Published:** 2025-07-02

**Authors:** QingYong Guo, Jinji Wang, Ru Chen, LiPing Hu, Wenqiang You

**Affiliations:** ^1^ Obstetrics & Gynecology, Fujian Maternity and Child Health Hospital College of Clinical Medicine for Obstetrics & Gynecology and Pediatrics, Fujian Medical University, Fuzhou, Fujian, China; ^2^ Medical Record Statistics, Fujian Maternity and Child Health Hospital College of Clinical Medicine for Medical Record Statistics, Fujian Medical University, Fuzhou, Fujian, China

**Keywords:** ovarian cancer, lymph node positivity, machine learning, XGBoost, prognosis

## Abstract

**Background:**

Ovarian cancer (OC) remains a highly lethal gynecological malignancy, often diagnosed at advanced stages with a poor prognosis. Lymph node involvement is a critical prognostic factor and significantly influences treatment planning. However, accurately predicting lymph node positivity remains challenging due to the disease’s heterogeneity and the limitations of traditional models in handling high-dimensional and imbalanced data.

**Methods:**

A retrospective analysis was conducted using the SEER database (2000–2021), including 26,844 OC patients with complete clinical information. We developed a machine learning model incorporating multiple algorithms, with XGBoost demonstrating superior performance. SMOTE was used to address class imbalance, and LASSO regression aided in selecting key predictors such as tumor size, histology, chemotherapy, and surgery. Model performance was assessed via accuracy, sensitivity, specificity, F1 score, and AUC, with external validation performed using an independent cohort from Fujian Provincial Maternity and Children’s Hospital.

**Results:**

The XGBoost model achieved an AUC of 0.98 (95% CI: 0.975–0.986) in the training set and 0.847 (95% CI: 0.823–0.871) in external validation. The model demonstrated high sensitivity and robust performance in identifying lymph node-positive cases. Tumor size ≥5 cm, histological subtype, and chemotherapy were key predictive features, with SHAP analysis identifying tumor size as the most influential factor.

**Conclusion:**

We present the first machine learning model specifically developed for predicting lymph node positivity in OC, validated across large, diverse cohorts. To facilitate clinical translation, we developed a free, user-friendly online calculator, which allows clinicians to quickly estimate lymph node positivity risk using patient-specific clinical parameters. This tool can be accessed at http://127.0.0.1:6818 and serves as a practical, evidence-based aid to support individualized treatment decisions and potentially improve patient outcomes. Future studies should integrate molecular data and broaden external validation to enhance generalizability.

## 1 Introduction

Ovarian cancer (OC) remains one of the most lethal gynecological malignancies worldwide, with high mortality rates largely due to late-stage diagnosis and metastasis at the time of presentation (1, 2). Despite advances in treatment, the five-year survival rate for patients diagnosed at advanced stages remains dismally low, emphasizing the urgent need for improved diagnostic and predictive tools. Lymph node involvement is a critical factor in OC prognosis, influencing treatment decisions and outcomes. However, accurately predicting lymph node positivity remains challenging due to the heterogeneity of the disease and the complexity of tumor biology (3, 4).

Current research on lymph node positivity in OC largely focuses on traditional statistical methods and clinicopathological parameters. Several studies have identified factors such as tumor grade, histological subtype, tumor size, and laterality as significant predictors of lymph node involvement (5–7). However, these approaches are often limited by the presence of missing data, multicollinearity among variables, and the lack of interpretability in high-dimensional settings. Additionally, traditional models tend to perform poorly in imbalanced datasets, where positive lymph node cases are relatively rare, resulting in limited sensitivity and predictive accuracy.

Machine learning (ML) techniques have emerged as powerful tools for predictive modeling, particularly in cases where complex, non-linear relationships exist among variables. In recent years, ML algorithms, such as gradient boosting, random forests, and XGBoost, have shown significant promise in the field of oncology, providing higher accuracy and robustness than traditional methods (8). ML models are particularly advantageous in handling high-dimensional data and imbalanced datasets, allowing for the inclusion of a wide range of clinical and demographic factors that may contribute to disease progression. Furthermore, methods like Synthetic Minority Over-sampling Technique (SMOTE) enable these models to improve performance on rare outcomes, such as lymph node positivity, by generating synthetic samples for underrepresented classes. This advancement is crucial in clinical applications, where the identification of high-risk patients can lead to more timely and targeted interventions.

Despite the potential of ML in OC prognosis, relatively few studies have applied these techniques specifically to predict lymph node positivity. Most existing models have focused on general survival outcomes or broad metastatic predictions without delving into lymph node involvement, which plays a unique role in OC spread and management (9, 10). Moreover, the lack of external validation in many studies raises concerns about the generalizability of these models across diverse patient populations. Therefore, there remains a significant gap in developing a robust, validated model for predicting lymph node involvement in OC, which could support clinicians in making more informed decisions on surgical staging and adjuvant therapy.

Our primary objective was to create a tool that could accurately assess lymph node positivity risk in OC patients, thereby assisting clinicians in identifying patients who may benefit from more aggressive staging and tailored treatment strategies. In addition to model development, we aimed to ensure external validity by testing our model on a separate dataset from a regional hospital, confirming its applicability across different clinical settings. Ultimately, this study provides a novel approach to OC prognosis, leveraging machine learning to address a clinically relevant question that has been challenging to answer with traditional methods. The resulting online calculator offers a practical, evidence-based tool for clinical use, supporting improved decision-making and potentially enhancing patient outcomes.

## 2 Method

### 2.1 Data sources and study population

**Figure 1:**
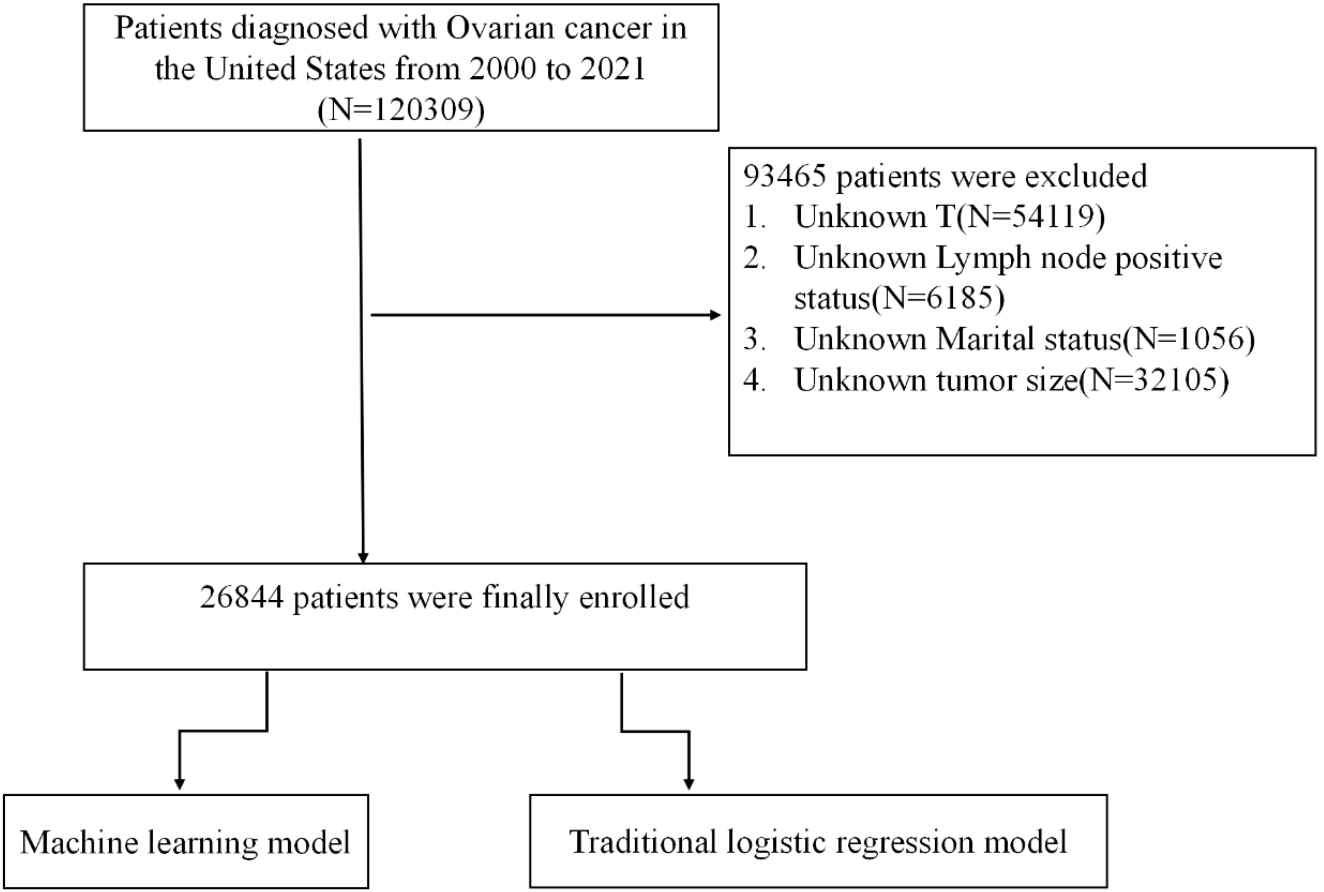
Study cohort selection flowchart.

This retrospective analysis leveraged data from the SEER database, spanning the years 2000 to 2021, to identify patients with a diagnosis of OC. The SEER database provides a comprehensive repository of clinical and demographic information at a national level, supporting population-based epidemiological research. Our initial cohort included 120,309 patients with OC. However, 93,465 individuals were excluded due to incomplete key data, specifically unknown T stage (N=54,119), indeterminate lymph node status (N=6,185), unspecified tumor size (N=32,105), and missing marital status (N=1,056), as visualized in **Figure 1**.

To assess the model’s robustness and external validity, we included a separate validation cohort comprising 550 patients diagnosed with OC at Fujian Provincial Maternity and Children’s Hospital. All patients in this external cohort had confirmed OC diagnoses and complete data for the predictors used in the model.

### 2.2 Identification of risk factors and model development

We applied the Least Absolute Shrinkage and Selection Operator (LASSO) regression approach to pinpoint significant clinical predictors for lymph node positivity in ovarian cancer. LASSO uses an L1 penalty, which selectively reduces certain coefficients to zero, isolating the most predictive variables. This method is particularly useful in high-dimensional data settings as it mitigates multicollinearity and provides a more interpretable model. Our analysis incorporated a variety of clinical factors, including patient age, race, marital status, household income, laterality, histology subtype, radiation therapy, surgery, chemotherapy, and tumor size. A 10-fold cross-validation strategy was employed to optimize the regularization parameter λ, minimizing prediction error and reducing the risk of overfitting. The final model incorporated critical predictors such as chemotherapy status, histology, surgical intervention, laterality, tumor grade, tumor size, marital status, race, and household income.

### 2.3 Model development and performance assessment

To forecast lymph node positivity, we developed and compared several machine learning algorithms, including Logistic Regression (LR), Random Forest (RF), Gradient Boosting Machine (GBM), Extreme Gradient Boosting (XGBoost), Naive Bayes (NB), and Classification and Regression Trees (CART). Logistic regression served as a linear baseline model, while Random Forest was selected for its robustness and capacity to manage non-linear relationships via an ensemble of decision trees. Both GBM and XGBoost employ iterative corrections to previous errors, with XGBoost offering enhanced regularization and computational efficiency. Naive Bayes was included for its simplicity and ability to handle high-dimensional data, whereas CART provided an interpretable decision tree structure for classification.

To address the imbalance in the dataset, where lymph node positivity was relatively rare, we applied the Synthetic Minority Over-sampling Technique (SMOTE) to the training data. SMOTE generates synthetic instances by interpolating between existing minority samples, thereby balancing the class distribution and improving the models’ sensitivity to rare outcomes.

We evaluated model performance using a set of comprehensive metrics: accuracy, sensitivity, specificity, F1 score, and the area under the receiver operating characteristic curve (AUC). Accuracy provided a measure of overall model correctness, while sensitivity and specificity quantified the model’s ability to correctly identify true positives and true negatives, respectively. The F1 score, particularly valuable in imbalanced datasets, balanced sensitivity and precision. AUC served as an indicator of the model’s discrimination capability across various thresholds. Additionally, precision-recall curves focused on the trade-off between precision and recall for the minority class, and calibration curves verified the agreement between predicted probabilities and actual outcomes. To further interpret model predictions, we utilized SHAP (SHapley Additive exPlanations) values to evaluate the contributions of individual predictors within the training data. This approach provided insights into the relative influence of each variable, enhancing our understanding of the underlying factors driving the model’s decision-making processes.

For external validation, we used an independent dataset of OC patients from Fujian Provincial Maternity and Children’s Hospital. This cohort was not involved in the training phase, allowing us to rigorously assess model generalizability across different clinical settings. The same evaluation metrics—accuracy, sensitivity, specificity, F1 score, and AUC—were applied to confirm the model’s robustness in an independent sample.

## 3 Result

A total of 26844 OC patients from the SEER database were included in this study, of whom 5795(22%) presented with lymph node positive, while 21049(78%) had no evidence of metastasis. The external validation cohort consisted of 550 patients diagnosed with OC at the Fujian Provincial Maternity and Children’s Hospital between 2018 and 2021, 118(21%) of whom had lymph node positive. Detailed cohort information is presented in **Table 1**. **Table 2** summarizes the baseline characteristics of OC patients with and without lymph node positive. Significant differences were observed between the groups in several key areas.

**Table 1:** Baseline characteristics of ovarian cancer patients from SEER database and external validation cohort.

Categories	Training set (N = 26844)	Validation set (N =550)	*P-* value
Age (years)			
mean (SD)	61.32 ± 15.06	63.15 ± 14.52	0.003
Race			0.236
Black	1549 (6%)	41 (7%)	
Other	3507 (13%)	73 (13%)	
White	21788 (81%)	436 (79%)	
Marital status			0.527
Divorced	2725 (10%)	43 (8%)	
Separated	13748 (51%)	284 (52%)	
Unmarried	255 (1%)	5 (1%)	
Widowed	5702 (21%)	125 (23%)	
Married	151 (1%)	4 (1%)	
Single	4263 (16%)	89 (16%)	
Household income			
<$60000	2365 (9%)	41 (7%)	
$60000 - $99,999	18791 (70%)	398 (73%)	
$100,000+	5688 (21%)	111 (20%)	
Laterality			0.866
Bilateral	7690 (29%)	157 (29%)	
Left	8116 (30%)	163 (30%)	
Paired site	3059 (11%)	69 (13%)	
Right	7979 (30%)	161 (29%)	
Histology recode			0.991
adenomas and adenocarcinomas	8348 (31%)	173 (31%)	
complex mixed and stromal neoplasms	866 (3%)	20 (4%)	
cystic, mucinous and serous neoplasms	13756 (51%)	280 (51%)	
epithelial neoplasms	1337 (5%)	26 (5%)	
germ cell neoplasms	673 (3%)	13 (2%)	
other	1233 (5%)	26 (5%)	
specialized gonadal neoplasms	539 (3%)	92 (2%)	
Grade			0.253
Grade I	1018 (4%)	15 (3%)	
Grade II	1764 (7%)	32 (6%)	
Grade III	4289 (16%)	76 (14%)	
Grade IV	2738 (10%)	54 (10%)	
Unknown	17035 (63%)	373 (67%)	
Radiation recode			0.648
None/Unknown	26354 (98%)	538 (98%)	
Yes	490 (2%)	12 (2%)	
Chemotherapy recode			0.256
No/Unknown	8205 (31%)	181 (33%)	
Yes	18639 (69%)	369 (67%)	
Surgery			0.717
None	4793 (18%)	102 (19%)	
Yes	22051 (82%)	448 (81%)	
Tumor Size (cm)			<0.001
<5	6382 (24%)	427 (78%)	
5-10	13067 (49%)	52 (9%)	
>10	7395 (28%)	71 (13%)	
Lymph node positive			0.981
No	21049 (78%)	432 (79%)	
Yes	5795 (22%)	118 (21%)	

SD, standard deviation.

**Table 2:** Baseline characteristics of ovarian cancer patients with and without lymph node positive in SEER database.

Categories	Without Lymph node positive (N = 21049)	Lymph node positive (N =5795)	*P-* value
Age (years)			
Mean (SD)	61.26 ± 15.11	61.54 ± 14.88	
Race			0.476
Black	1196 (6%)	353 (6%)	
White	17094 (81%)	4694 (81%)	
Other	2759 (13%)	748 (13%)	
Marital status			0.381
Divorced	2122 (10%)	603 (10%)	
Separated	10791 (51%)	2957 (51%)	
Unmarried	207 (1%)	48 (1%)	
Widowed	4488 (21%)	1214 (21%)	
Married	126 (1%)	25 (1%)	
Single	3315 (16%)	948 (16%)	
Household income			0.313
<$60000	4426 (21%)	1262 (22%)	
$60000 - $99,999	14748 (70%)	4043 (70%)	
$100,000+	1875 (9%)	490 (8%)	
Laterality			<0.001
Bilateral	5883 (28%)	1807 (32%)	
Left	6492 (31%)	1624 (28%)	
Paired site	2344 (11%)	715 (12%)	
Right	6330 (30%)	1649 (28%)	
Histology recode			<0.001
adenomas and adenocarcinomas	6708 (32%)	1640 (28%)	
complex mixed and stromal neoplasms	686 (3%)	180 (3%)	
cystic, mucinous and serous neoplasms	10568 (50%)	3188 (55%)	
epithelial neoplasms	1037 (5%)	300 (5%)	
germ cell neoplasms	546 (2%)	127 (2%)	
specialized gonadal neoplasms	539 (3%)	92 (2%)	
other	965 (5%)	268 (5%)	
Grade			0.017
Grade I	794 (3.77%)	224 (3.86%)	
Grade II	1344 (6.38%)	420 (7.24%)	
Grade III	3349 (15.91%)	940 (16.22%)	
Grade IV	2108 (10.01%)	630 (10.87%)	
Unknown	13454 (63.93%)	3581 (61.81%)	
Radiation recode			0.254
None/Unknown	20654 (98%)	5700 (98%)	
Yes	395 (2%)	95 (2%)	
Chemotherapy recode			<0.001
No/Unknown	6624 (31%)	1581 (27%)	
Yes	14425 (69%)	4214 (73%)	
Surgery			0.006
None	3688 (18%)	1105 (19%)	
Yes	17361 (82%)	4690 (81%)	
Tumor Size (cm)			0.339
<5	5029 (24%)	1353 (24%)	
5-10	10264 (49%)	2803 (48%)	
>10	5756 (27%)	1639 (28%)	

SD, standard deviation.

**Figure 2:**
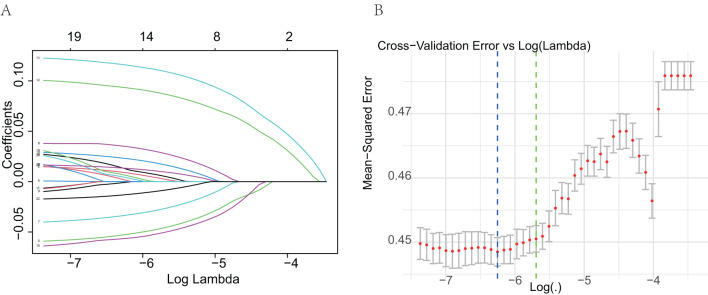
Selection of predictive variables using Lasso logistic regression. **(A)** Lasso coefficient profiles of the variables. Each curve represents a predictor; the X-axis corresponds to the log-transformed regularization parameter (lambda), and the Y-axis shows the coefficient values. As the penalty increases, many coefficients shrink to zero. **(B)** Selection of optimal lambda via 10-fold cross-validation. The plot shows the mean squared error for each value of lambda. The dotted line indicates the lambda with the minimum cross-validation error.

**Figure 3:**
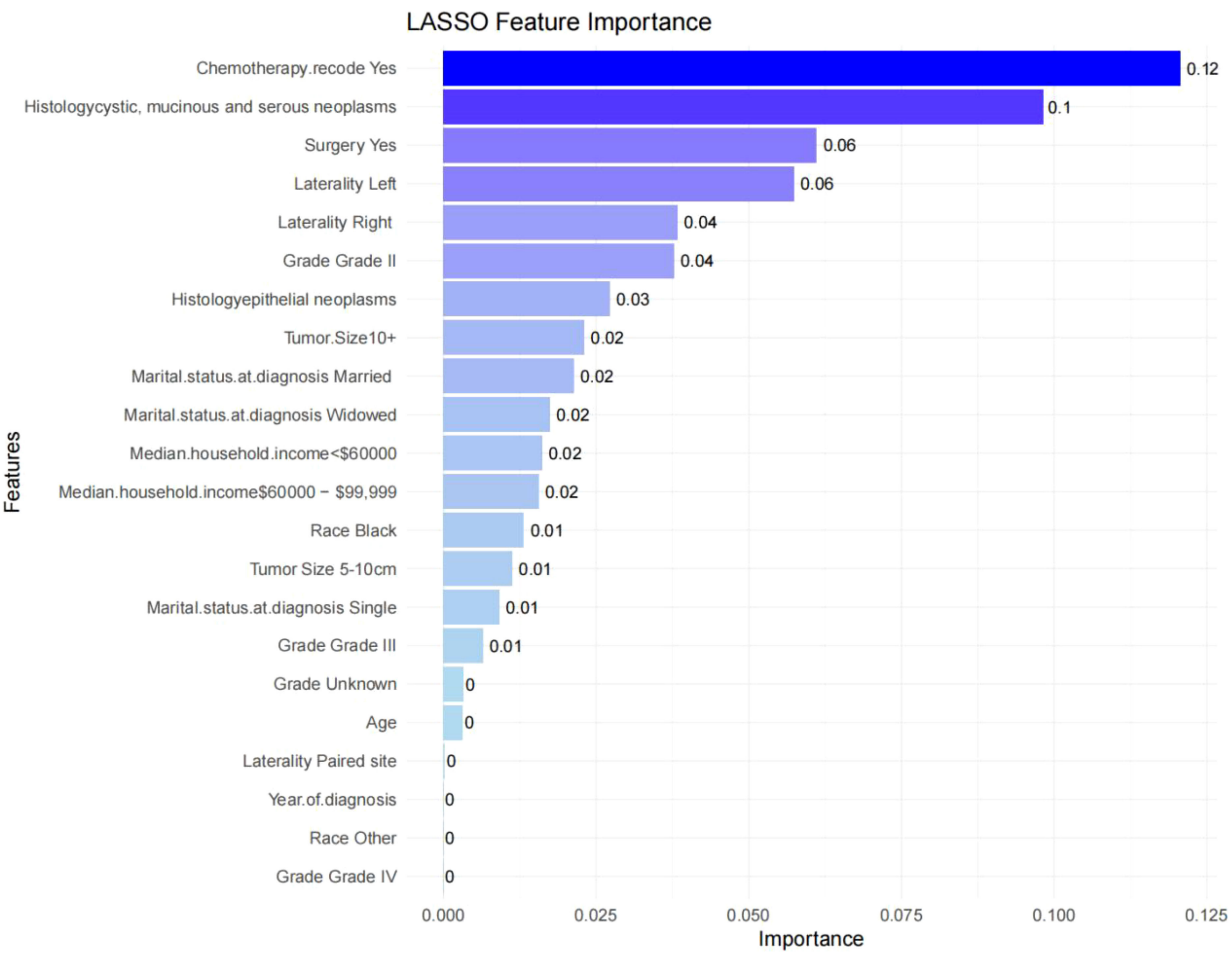
Feature importance in the final XGBoost model. Feature importance scores ranked by weight. Features such as chemotherapy, histology, and surgery show high importance in predicting lymph node metastasis.

The final predictive model was refined to include nine key variables, chosen for their consistency along the regularization path and their substantial impact on reducing cross-validation error. These variables demonstrated robust predictive power in assessing metastasis risk among ovarian cancer patients (**Figures 2A, B**). Notably, the selected features likely encompass several critical factors highlighted in the feature importance plot (**Figure 3**), including chemotherapy, histology, surgery, and laterality—factors well-established as pivotal in the prognosis and progression of ovarian cancer.

**Figure 4:**
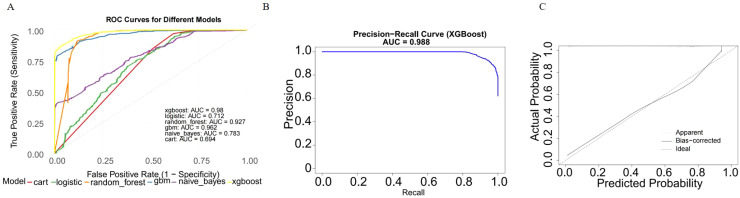
Model performance of XGBoost on the training dataset. **(A)** ROC curve of the XGBoost model. The model achieved an AUC of 0.98 (95% CI: 0.975–0.986), indicating excellent diagnostic performance. **(B)** Precision-recall curve. The AUC for the PR curve was 0.988, reflecting strong performance on the imbalanced dataset. **(C)** Calibration curve. Predicted probabilities closely matched observed outcomes, indicating good model.

**Figure 5:**
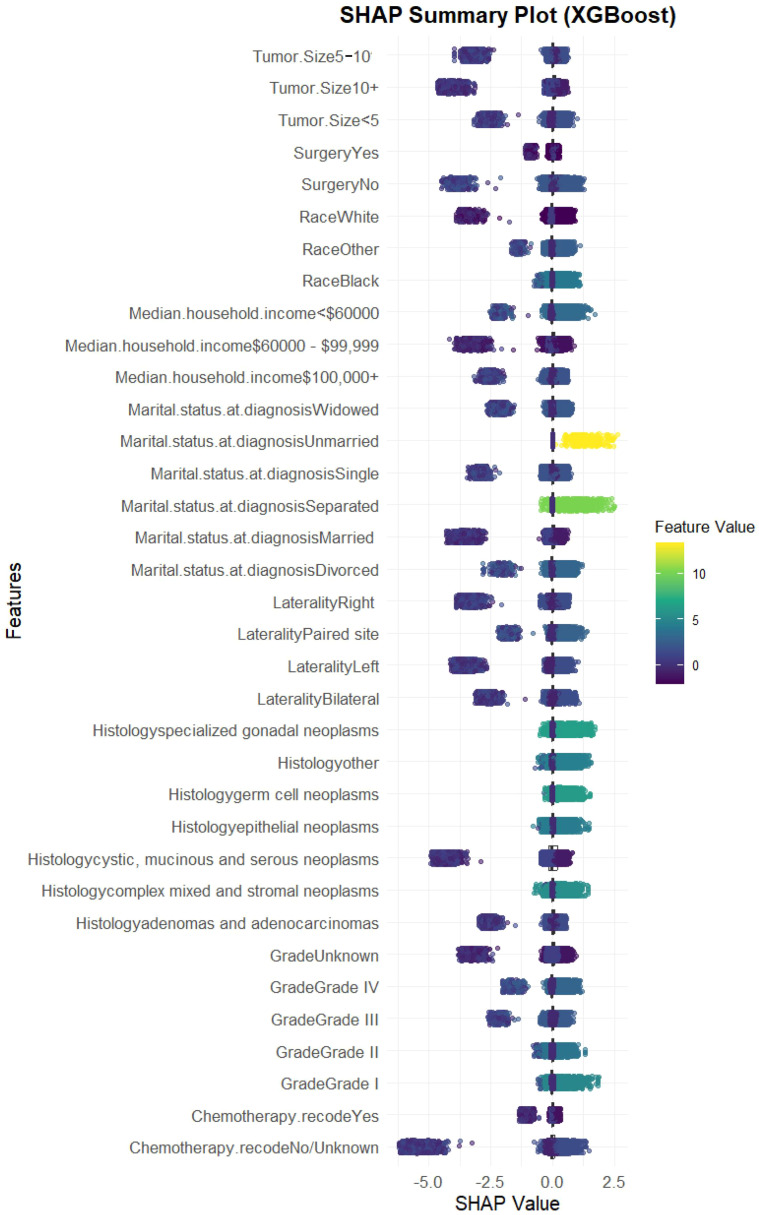
SHAP summary plot for the XGBoost model. SHAP values showing the impact of each feature. Tumor size ≥5 cm emerged as the most influential variable, followed by laterality, chemotherapy, and histology.

We evaluated the performance of seven machine learning algorithms to predict lymph node positivity in ovarian cancer, assessing metrics such as accuracy, precision, recall, F1 score, and AUC. Consistent with prior studies, models trained with oversampling techniques outperformed those using undersampling. Detailed performance metrics for each model are provided in **Table 3**. Among the oversampled models, all achieved an AUC above 0.6, with XGBoost delivering the highest performance, reaching an AUC of 0.98 (95% CI: 0.975–0.986) on the training set (**Figure 4A**). Comparison of AUC values between XGBoost and traditional logistic regression showed that XGBoost offered significantly enhanced diagnostic accuracy and predictive power. The precision-recall curve for XGBoost demonstrated an AUC of 0.988, underscoring its strong capacity to handle the imbalanced dataset, where lymph node-positive cases are underrepresented (**Figure 4B**). The calibration curve for XGBoost showed excellent agreement between predicted probabilities and observed outcomes, indicating robust calibration (**Figure 4C**). The SHAP summary plot illustrates the influence of various features in the XGBoost model’s predictions for lymph node positivity in ovarian cancer patients (**Figure 5**), with tumor size ≥5 cm emerging as the most impactful factor. Larger tumor sizes were associated with a significantly higher likelihood of lymph node positivity, while smaller tumors correlated with lower risk.

**Table 3:** Performance metrics of machine learning models for predicting lymph node positive in ovarian cancer patients.

Model	Accuracy	Sensitivity	Specificity	F1_Score	AUC
Training set					
Logistic	0.624	0.02	0.99	0.039	0.712
Random forest	0.64	0.047	1	0.089	0.927
Gbm	0.694	0.364	0.895	0.473	0.962
Xgboost	0.879	0.787	0.935	0.831	0.982
Naive bayes	0.623	0	1	0.676	0.783
Cart	0.644	0.212	0.905	0.31	0.694
Validation set					
Xgboost	0.711	0.286	0.968	0.427	0.847

**Figure 6:**
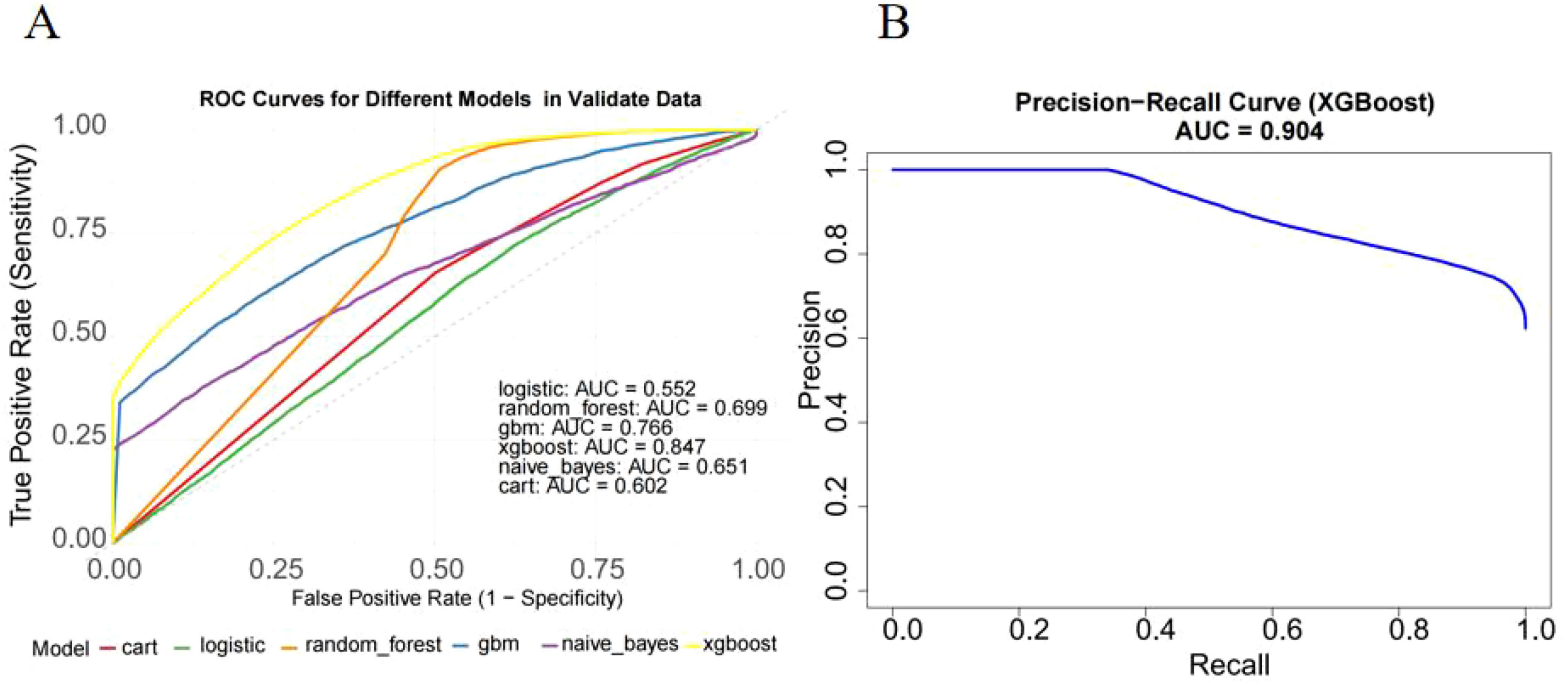
External validation of the XGBoost model on the independent cohort. **(A)** ROC curve on external validation dataset. The model achieved an AUC of 0.847 (95% CI: 0.823–0.871), confirming strong generalization ability. **(B)** Precision-recall curve showed AUC of XGBoost(0.904).

In external validation, XGBoost continued to perform well, achieving an AUC of 0.847 (95% CI: 0.823–0.871) (**Figure 6A**). The model displayed a balanced performance, with relatively high sensitivity that enhances its ability to identify lymph node-positive cases, although specificity was moderate, suggesting some false positives. Overall, the model achieved a high F1 score (0.866) (**Table 3**) and AUC (**Figure 6B**), confirming its efficacy on validation data.

**Figure 7:**
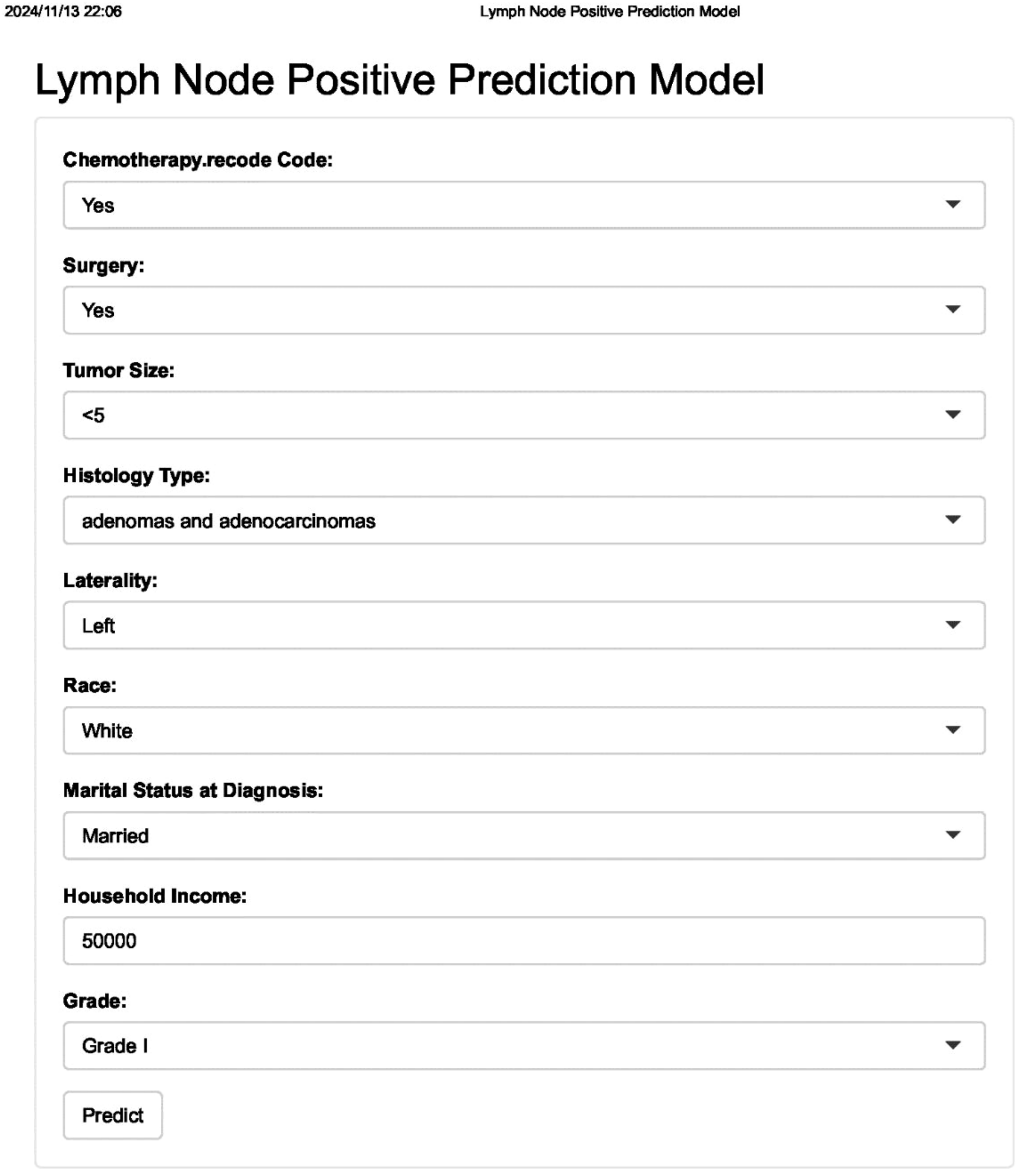
Web-based interface for clinical use. The tool allows clinicians to input patient data and receive immediate risk estimates for lymph node positivity.

Finally, we developed an online calculator to assess the risk of lymph node positivity in ovarian cancer patients, which can be used in clinical settings (**Figure 7**). The tool is available at http://127.0.0.1:6818. By clicking on the website and entering the corresponding clinical variables, a score will be generated, allowing the evaluation of the risk of lymph node metastasis.

## 4 Discussion

This study developed and validated a machine learning model to predict lymph node positivity in OC patients, addressing an important clinical challenge. Our primary finding is that machine learning, particularly the XGBoost algorithm, demonstrates high predictive accuracy, sensitivity, and generalizability in assessing lymph node involvement. Through comprehensive evaluation using multiple metrics—accuracy, sensitivity, specificity, F1 score, and AUC—we confirmed the robustness of our model both in the SEER cohort and the external validation dataset. This tool, available as an online calculator, provides a practical solution for clinicians to assess lymph node positivity risk, potentially guiding treatment planning and surgical staging decisions.

Previous studies have predominantly relied on conventional statistical models and clinicopathological parameters to predict lymph node involvement in OC. While factors such as tumor grade(Grade I to IV or well-differentiated to undifferentiated), histological subtype, tumor size, and laterality have been consistently identified as predictors, traditional models are often constrained by their limited ability to handle high-dimensional data, missing values, and multicollinearity (11–13). Moreover, the predictive performance of these models typically suffers in imbalanced datasets, which is a common issue in studies on lymph node positivity where positive cases are rare. For example, logistic regression models may struggle with imbalanced data, leading to reduced sensitivity and an inability to generalize well across diverse patient populations.

In contrast, our study leveraged advanced machine learning techniques, which are inherently more suited to handling complex, high-dimensional, and imbalanced datasets. By employing XGBoost, we addressed the non-linear relationships between clinical features and lymph node positivity, which conventional models may overlook. Moreover, applying SMOTE allowed us to mitigate the effects of class imbalance, thereby enhancing the model’s sensitivity and overall predictive power. Unlike previous models that primarily focus on survival outcomes or broad metastatic risk, our approach specifically targets lymph node involvement, addressing a clinically relevant aspect of OC progression that can significantly influence treatment decisions.

The final XGBoost model incorporated nine key predictors, including tumor size, histology, chemotherapy, surgery, laterality, tumor grade, marital status, race, and household income. Among these, tumor size emerged as the most influential factor, with larger tumors (≥5 cm) being strongly associated with lymph node positivity. This finding aligns with existing literature, as larger tumors are more likely to facilitate lymphatic spread. Other predictors, such as histological subtype and laterality, also showed significant associations with lymph node involvement, consistent with known biological mechanisms in OC (14–16).

The SHAP analysis provided further insight into the model’s decision-making process, highlighting how each variable contributed to the risk assessment. For instance, chemotherapy and surgery were notable predictors, suggesting that patients who had undergone these treatments might exhibit different lymph node involvement risks due to alterations in tumor biology or immune response. Such detailed interpretability not only enhances our understanding of OC progression but also provides clinicians with a transparent tool for assessing individual patient risks.

Our model addresses a critical gap in OC management by providing an evidence-based tool for predicting lymph node involvement, thereby aiding in personalized treatment planning. Accurate lymph node assessment is crucial for determining the extent of surgical staging, selecting patients for lymphadenectomy, and identifying those who may benefit from adjuvant therapy. Traditional methods for lymph node assessment, such as sentinel lymph node biopsy or extensive lymphadenectomy, are invasive and associated with morbidity. By offering a non-invasive prediction tool, our model enables more targeted interventions, potentially reducing unnecessary procedures and their associated complications. Moreover, the model’s online calculator offers an accessible platform for clinicians, allowing them to input readily available clinical data and receive an immediate risk assessment. This practical tool can be particularly valuable in resource-limited settings where comprehensive imaging or molecular testing may not be feasible.

A major strength of our study is that it represents the first machine learning-based model developed specifically to predict lymph node positivity in ovarian cancer. While previous research has focused on general survival outcomes or broad metastatic predictions, none have targeted lymph node involvement—a critical factor influencing OC treatment decisions and prognosis. By addressing this clinically relevant issue, our study fills an important gap in OC research, providing a novel and specialized tool for clinicians. In addition, we utilized a large, nationally representative dataset (SEER) for model training, combined with external validation on an independent cohort from Fujian Provincial Maternity and Children’s Hospital. This extensive approach enhances the generalizability of our findings, addressing a common limitation in predictive modeling studies that often lack validation across diverse populations. Furthermore, our application of XGBoost and SMOTE exemplifies the utility of machine learning in handling complex, imbalanced datasets, a common challenge in the clinical oncology setting. Another unique advantage of our study is the interpretability of the model through SHAP values, which allows clinicians to understand how each predictor contributes to the overall prediction. This transparency fosters trust in the model’s outputs and provides insight into the underlying factors associated with lymph node positivity in OC, which traditional models rarely achieve.

However, despite these strengths, several limitations of this study should be acknowledged. First, as a retrospective analysis based on the SEER database, our study may be subject to inherent selection bias and residual confounding, which cannot be fully eliminated. Second, although the SEER database provides a large and representative sample, it lacks important clinical details such as FIGO staging, genetic profiles (e.g., BRCA1/2 mutation status), molecular markers, and information on residual disease or chemotherapy regimens. These factors are known to influence lymph node metastasis risk and treatment decisions and would further enhance the predictive accuracy and clinical utility of the model if available. Third, while the external validation cohort showed good generalizability, it was limited to a single center in China. Validation in broader, multi-center, and international settings is necessary to confirm the model’s applicability across different populations and healthcare systems. Finally, although our model demonstrated high sensitivity in identifying lymph node-positive cases, its moderate specificity may lead to some false-positive predictions. While this trade-off may be acceptable in clinical scenarios where prioritizing high-risk detection is critical, future versions of the model should aim to optimize the balance between sensitivity and specificity to minimize unnecessary interventions.

## 5 Conclusion

In conclusion, our study marks a significant advancement as the first machine learning model specifically designed to predict lymph node positivity in ovarian cancer. Through innovative machine learning techniques and rigorous external validation, we provide a robust, interpretable, and practical tool that enhances clinical decision-making in OC. This model offers clinicians an evidence-based approach to assess lymph node positivity risk, aiding in surgical and treatment planning and ultimately contributing to improved patient outcomes. Future research should build on these findings, incorporating molecular data and validating the model in diverse clinical populations to further refine its utility in ovarian cancer care.

## Data Availability

The original contributions presented in the study are included in the article/supplementary material. Further inquiries can be directed to the corresponding authors.
